# First record of *Holothuria (Metriatyla) scabra* Jaeger, 1833 (Echinodermata: Holothuroidea) from the coastal waters of the United Arab Emirates

**DOI:** 10.7717/peerj.5555

**Published:** 2018-09-28

**Authors:** Fadi Yaghmour, Brendan Whittington-Jones

**Affiliations:** 1 Scientific Research Department, Environment and Protected Areas Authority, Kalba, Sharjah, United Arab Emirates; 2 Scientific Research Department, Environment and Protected Areas Authority, Sharjah, Sharjah, United Arab Emirates

**Keywords:** Bêche-de-mer, Endangered, Echinodermata, Holothuroidea, *Holothuria (Metriatyla) scabra*, United Arab Emirates, Sea cucumber, Gulf of Oman, Khor Kalba, Species identification

## Abstract

The presence of an endangered and economically valuable species of sea cucumber, *Holothuria* (*Metriatyla*) *scabra* Jaeger, 1833, was investigated in the Alqurm Wa Lehhfaiiah Protected Area near the city of Kalba in the Emirate of Sharjah. Sea cucumber specimens were collected, and identification was first conducted using morphological keys. *H. scabra* identification was confirmed through microscopic observation of ossicles. Though this species is known to occur in other regions along the Gulf of Oman, this paper represents the first published record of *H. scabra*, in the coastal waters of the United Arab Emirates.

## Introduction

Many marine and terrestrial invertebrates are understudied and also suffer higher rates of threat of extinction than other taxonomic groups like mammals (McKinney, 1999; Cardosoa et al., 2011). Invertebrates are also frequently neglected in biodiversity conservation policies (McKinney, 1999; Cardosoa et al., 2011). Holothurians, hereafter referred to as “sea cucumbers,” are a class of echinoderm that consist of six orders and nearly 1,400 species (Dabbagh et al., 2012; WoRMS, 2018). They play an important ecological role and present an economic opportunity. Ecologically, they are prolific recyclers of benthic organic matter through the consumption of sediments, organic detritus and seagrasses, and produce fecal pellets (Dar & Ahmad, 2006; Wolkenhauer et al., 2010). Economically, sea cucumbers are subject to significant international commercial trade (Kithakeni & Ndaro, 2002; Lovatelli et al., 2004; Al-Rashdi, Al-Busaidi & Al-Rassadi, 2007; Hamel et al., 2013; Purcell, 2014). Global overharvesting has resulted in the declining of wild stocks (Kithakeni & Ndaro, 2002; Al-Rashdi, Al-Busaidi & Al-Rassadi, 2007; Wolkenhauer et al., 2010; Dabbagh et al., 2012; Hamel et al., 2013). This challenge to biodiversity and habitat conservation requires distribution ranges to be adequately recorded to inform appropriate management measures. In particular, sandfish, *Holothuria (Metriatyla) scabra* Jaeger, 1833 is heavily commercially exploited from coastal near-shore waters for consumption as bêche-de-mer (Hamel et al., 2013; Bastami et al., 2012). *H. scabra* is distributed in the tropical and subtropical areas of the Western Indo-Pacific Ocean between latitudes 30°N and 30°S (Dabbagh et al., 2012; Massin et al., 2009; Hamel et al., 2013; Bastami et al., 2012).

*Holothuria scabra* has been described as occurring naturally in the waters of Saudi Arabia (Price, 1982), Oman (Al-Rashdi, Al-Busaidi & Al-Rassadi, 2007), and Iran (Dabbagh et al., 2012). The United Arab Emirates is one of the principal Middle Eastern exporters of *H. scabra*, despite the species not being formally recorded to occur in the country (Al-Rashdi, Al-Busaidi & Al-Rassadi, 2007; Dabbagh et al., 2012). The primary source of this exported stock is importation from neighboring Oman (Al-Rashdi, Al-Busaidi & Al-Rassadi, 2007). In this context, it was determined by the Environment and Protected Areas Authority of Sharjah (EPAA) that the identification of conspicuously distributed sea cucumbers within the mangal channels of the Alqurm Wa Lehhfaiiah Protected Area merited investigation. Here, we report the first record of *H. scabra* from the coastal waters of the United Arab Emirates.

## Materials and Methods

### Study site

**Figure 1:**
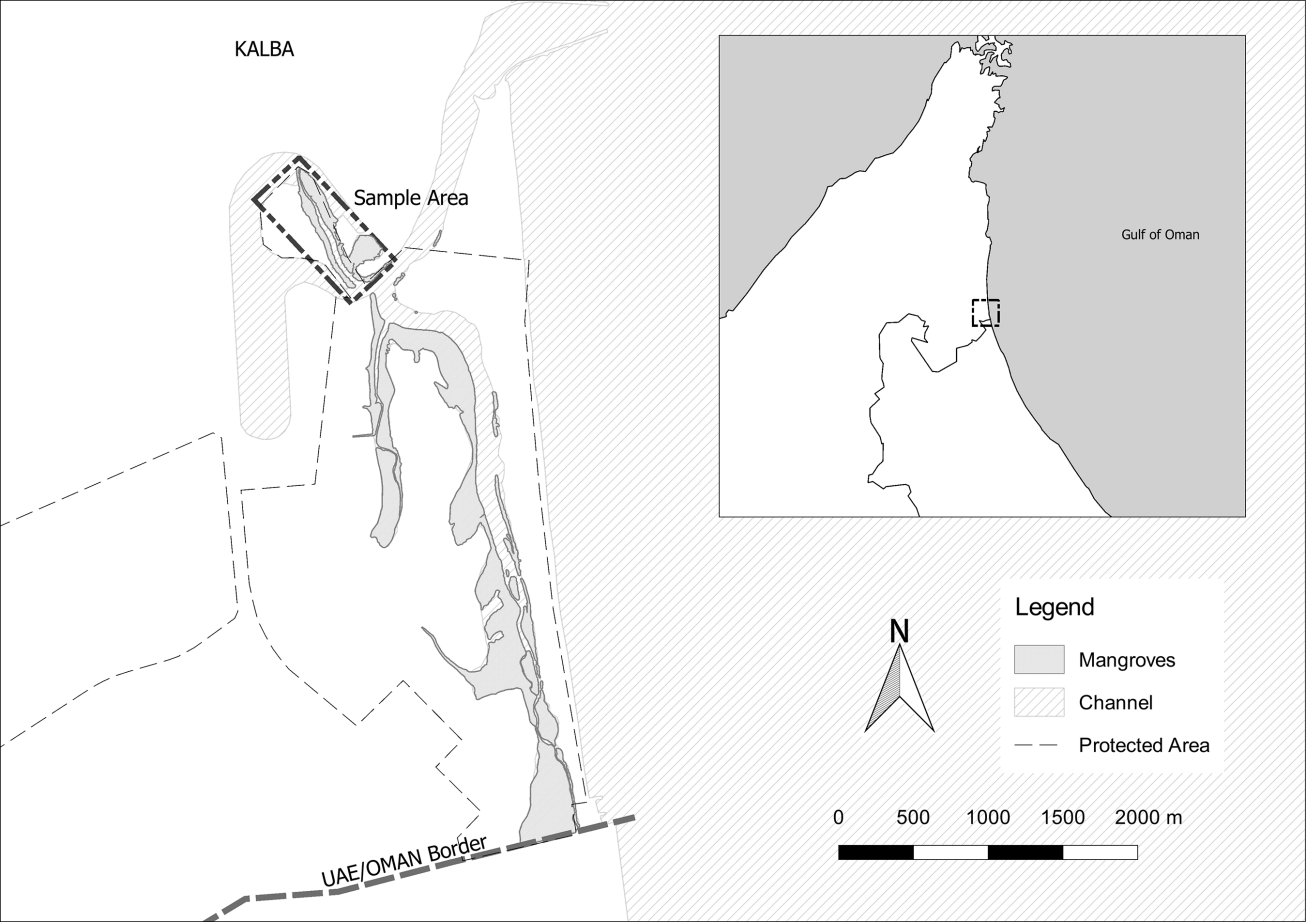
Sea cucumber sampling site in the Alqurm Wa Lehhfaiiah Protected Area of the city of Kalba, Sharjah, United Arab Emirates. Map by John Pereira.

Alqurm Wa Lehhfaiiah Protected Area is situated in the Gulf of Oman (Fig. 1). A six km^2^ shallow tidal inlet (25.028223 N, 56.368909 E) leads to a series of saline (33–44 ppt) channels fringed with mangrove woodlands of *Avicennia marina*. The benthic substrate of channels comprises sandy bottoms, seagrass beds, and rocky reefs with sparse coral colonies. Subtidal and intertidal mudflats lie adjacent to the surrounding terrestrial habitat of saltmarsh (with associated halophytic plant communities), low sand dunes and sandy beach.

### Specimen sampling

Five sea cucumber specimens were collected during low tide from exposed sandy bottom substrates and seagrass beds on March 3rd, 2017 (Fig. 1). Gross morphology of the five specimens was noted. Tissue samples (two cm^2^) of the dorsal body wall/dorsal papillae and ventral body wall/tube feet were collected from three specimens, prior to release. Samples were put in plastic jars with approximately five ml of household bleach.

### Sample analysis

Tissue samples were left in household bleach until the body wall had completely dissolved, leaving only the ossicles at the bottom (Ong, Wirawati & Wong, 2016). Using a pipette, the ossicles were washed three times with distilled water and transferred onto a microscope slide where they were examined using an Olympus CX23 compound microscope.

## Results

**Figure 2:**
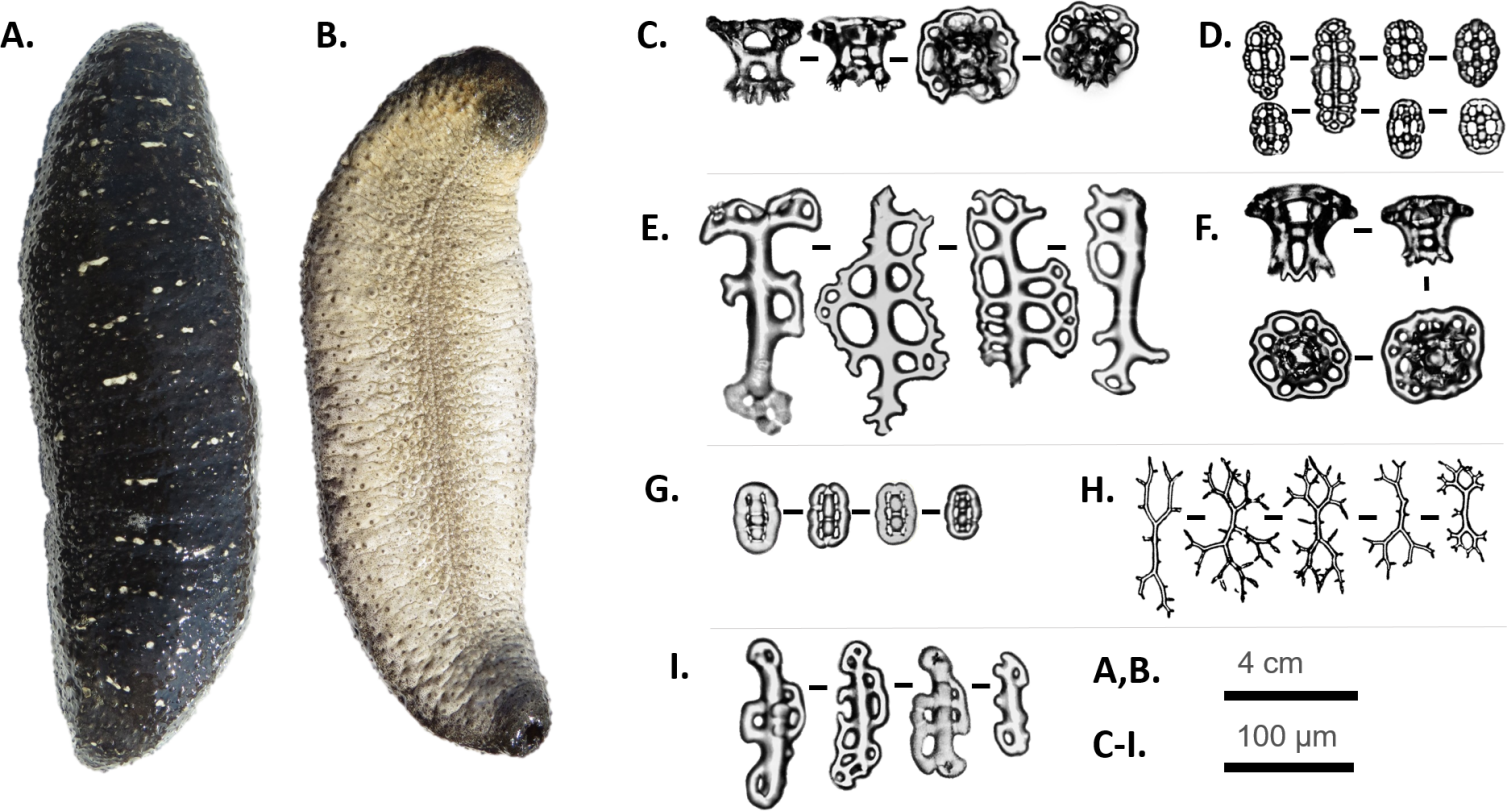
*Holothuria (Metriatyla) scabra* Jaeger, 1833. Live specimen (A, B) and ossicles (C–I): (A) dorsal view of living *H. scabra* specimen (22 cm); (B) ventral view of living *H. scabra* specimen (22 cm); (C) tables from dorsal body wall and dorsal papillae; (D) buttons from dorsal body wall and dorsal papillae; (E) perforated rods of dorsal papillae; (F) tables of ventral body wall and tube feet; (G) buttons of ventral body wall and tube feet; (H) branched rods of ventral body wall and tube feet; (I) perforated rods of ventral tube feet. Scale bars = 4 cm (A, B), 100 μm (C–I).

**SYSTEMATICS**
**Phylum ECHINODERMATA** Bruguière, 1791
**Class HOLOTHUROIDEA** deBlainville, 1834
**Order HOLOTHURIIDA** Miller, Kerr, Paulay, Reich, Wilson, Carvajal & Rouse, 2017
**Family HOLOTHURIDAE** Burmeister, 1837
**Genus *Holothuria*** Linnaeus, 1767
**Subgenus *Metriatyla*** Rowe, 1969
***Holothuria (Metriatyla) scabra*** Jaeger, 1833 (Fig. 2)

### Morphology

Specimens predominantly characterized by dark gray to black coloration with fine transverse white streaks and transverse wrinkles on the dorsal surface (Fig. 2A). Bodies gritty with arched dorsal surface and flat ventral surface. Ventral surface noticeably lighter than dorsal surface, appearing pale gray with small black dots where tube feet are located (Fig. 2B). Mouth is ventral, surrounded by 20 short tentacles.

Ossicles of dorsal body wall of *H. scabra* consist of tables (Fig. 2C) and buttons (Fig. 2D). Tables are rare, small and stout about 63 μm long, discs with undulating rims, 80 μm in diameter. Discs are perforated by one central hole and one circle of 8–16 peripheral holes of various sizes. Spire consists of four pillars bridged together by a single cross-beam. Spire ends in crown of blunt spines, perforated by one central hole. Crown never as wide as table disc but also perforated in the middle. Buttons are nodulous with mean length of 41.5 μm. Buttons perforated by three to four pairs of holes, with some larger buttons comprising five to seven pairs of holes (Fig. 2D). Dorsal papillae consist of few perforated rods, 128 μm in length (Fig. 2I).

Ventral surface tables similar to dorsal tables, 49.3 μm long with a mean disc width of 70.5 μm (Fig. 2E). Ventrally, buttons are very numerous and nodulous, 43.1 μm long (Fig. 2F). Observation of ossicles of tentacles and ventral body wall showed small branched rods and small smooth rods (Fig. 2G). Rods are 116 μm in length. Tube feet have perforated rods, 124 μm long (Fig. 2H).

## Discussion

There is considerable variation in the color morphs of *H. scabra*. These include greenish gray (Uthicke & Benzie, 1999), and brown and black (Massin et al., 2009) specimens. The dorsal body of the *H. scabra* specimens observed in this study is typical of those found in the western Indo-Pacific region, having a gray–black dorsal body wall interrupted with white–yellow transverse streaks (Al-Rashdi, Al-Busaidi & Al-Rassadi, 2007; Samyn & Vanden, 2000; Kithakeni & Ndaro, 2002; Massin et al., 2009; James, 2001). In our specimens branched rods were observed among the ossicles, which according to (Dabbagh et al., 2012), was only described in specimens in Qeshm Island (Iran) and in Madagascar (Cherbonnier, 1988).

## Conclusion

*Holothuria scabra* populations have been globally overexploited by intensive commercial extraction, to a state where they are listed as Endangered by the International Union for the Conservation of Nature. Most heavily exploited sea cucumber populations suffer rapid declines in their abundance and population densities with the onset of commercial exploitation (Conand, 1997; Uthicke & Conand, 2005; Al-Rashdi & Claereboudt, 2010). This trend was also observed in the fishing grounds in Mahout Bay, Oman (Al-Rashdi, Al-Busaidi & Al-Rassadi, 2007). Despite these threats to the persistence of this species, and the economic significance, sea cucumbers remain rarely studied in the region. In this study, we report *H. scabra* for the first time in the United Arab Emirates. Further research within the coastal waters under the jurisdiction of Sharjah’s EPAA will aid in determination of *H. scabra* range in the UAE.

## Additional Information and Declarations

### Data Availability

The following information was supplied regarding data availability: The research in this article did not generate any data or code as the paper focuses on a morphologic description.
